# Mori–Tanaka Formalism-Based Method Used to Estimate the Viscoelastic Parameters of Laminated Composites †

**DOI:** 10.3390/polym12112481

**Published:** 2020-10-26

**Authors:** Mostafa Katouzian, Sorin Vlase

**Affiliations:** 1 Department Maschinenwesen, Technical University of Munich, 85748 München, Germany; m.katouzian@web.de; 2 Department of Mechanical Engineering, Transilvania University of Brașov, 500036 Brasov, Romania; 3 Romanian Academy of Technical Sciences, 010071 Bucharest, Romania

**Keywords:** fibrous composite, homogenization method, Mori–Tanaka theory, Schapery’s equation, constitutive law

## Abstract

The paper establishes the mechanical properties of a viscoelastic composite material reinforced with fibers, where the fiber is transverse isotropic and the matrix is isotropic (a common case met in engineering practice). A computation method using the Mori–Tanaka mean field method has been developed in order to apply on viscoelastic materials. Using this procedure, the time-dependent response of a viscoelastic composite material can be determined. Schapery’s nonlinear constitutive equation is also used in the compliance matrix determination of the composite material under investigation. Nonlinearity factors were determined by creep tests at different values of stresses and temperatures and for different materials, based on the least squares method. The results obtained experimentally and their comparison with the theoretically obtained values show a good agreement between experiment and calculation.

## 1. Introduction

The viscoelastic response of a composite material, consisting of two or more components, is determined by the elastic/viscoelastic properties of the components. The calculation of these properties, based on various mathematical models, using fundamental theorems from the theory of elasticity, variational methods, averaging methods, homogenization theory, etc., was made in a series of papers for biphasic materials [1,2,3,4,5,6]. However, we must mention that there is little work in the literature dedicated to the study of viscoelastic materials subjected to high temperatures. In most of these works, the basic hypothesis considered was that, the constituent materials of the composite are isotropic. In practice, however, there are many cases in which one of the materials has anisotropic properties. In this case, the classical model established for predicting the property of the composite no longer works. However, there are few works in the literature when the reinforcement material has anisotropic or transverse isotropic properties. The problem is, therefore, to establish new relations valid for determining the global properties in these new circumstances. As an example, treated in the paper, a composite will be considered that is reinforced with graphite/epoxy fibers, where the fibers represent a material with anisotropic properties. The reinforcing parallel fibers are considered long enough that the end effects can be neglected.

The calculation method will consider a representative cylindrical element volume (RVE), having the fibers arranged along the axis of the cylinder. The cross section of this cylinder is large enough in relation to the dimensions of the fibers, so that averaging operations can be performed unaffected by the small non-homogeneities in the structure and by the arrangement of the fibers. The fibers will be considered continuous along the entire length RVE [7,8]. It will be hypothesized that the composite is considered transversely isotropic and homogeneous.

In these hypotheses, we use a mathematical model by which the elastic/viscoelastic properties of the material, will be determined by upper and lower bounds of the properties of the components, which are considered to be known.

Using Hill’s theory [9,10,11], one may obtain the bounds on bulk modulus, the longitudinal Young’s modulus and the Poisson’s ratio. These bounds are determined in terms of the volume ratios of fiber *v_f_* and matrix *v_m_*.

For a reinforced composite with cylindrical fibers, the upper and lower bounds on the effective elastic moduli have been calculated for a geometry of hexagonal fibers array by Hashin [12,13,14,15].

A new direction of research represents the gradual change of the fiber orientation and the study of material at high stress level and temperature. A better model is presented in the following, where exact estimations for some engineering constants are determined.

In the last few years a series of papers studied the viscoelastic creep response of different material subjected to different loads and temperatures.

The creep behavior of linear viscoelastic materials subjected to a combined tension and torsion loading is presented in [16]. The creep response is expressed using fractional-exponential functions. Experimental tests have validated the model for HDPE and organic glass.

All the phases of creep, primary, secondary, tertiary and rupture, are analyzed in [17], based on a nonlinear viscoelastic creep constitutive formula. The parameters of this model are analyzed and evaluated. Other papers studied different aspects of the creep response of different materials [18,19,20,21,22,23,24].

## 2. Mathematical Model

**Figure 1:**
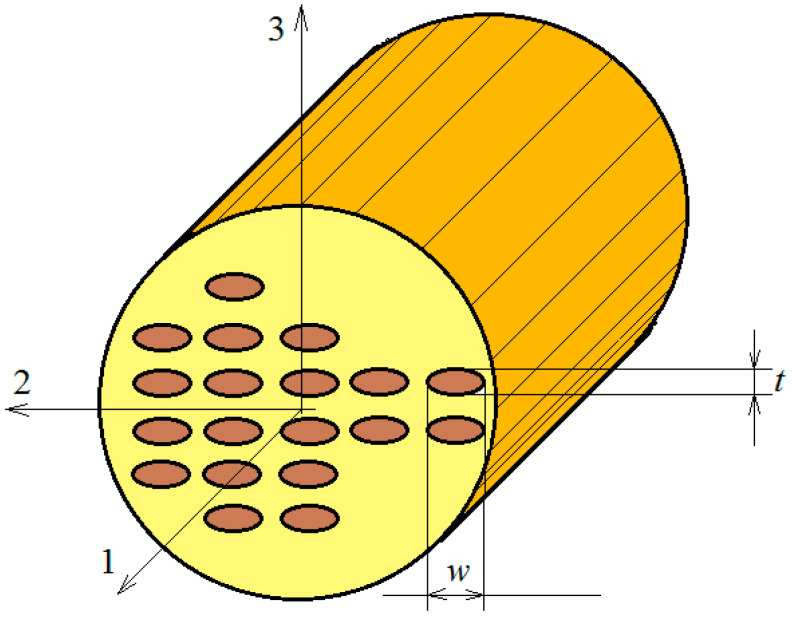
Reinforced composite with aligned elliptic fibers.

To obtain the elastic/viscoelastic coefficients of the biphasic composite, reinforced with long, anisotropic fibers, the Mori–Tanaka theory will be used [25]. For this, RVE as defined in the Introduction section of this paper, will be used, consisting of an epoxy matrix with viscoelastic behavior, reinforced with monotonously aligned and parallel fibers, uniformly distributed within the matrix. Considering the above, the composite obtained will be regarded as orthotropic. If, however, the elliptical cylinders fibers are randomly oriented, the composite body under consideration will be viewed as transverse isotropic. The elliptical fibers are defined by the ratio α=t/w (see Figure 1). If the fibers are circular, this ratio is 1.

Eshelby’s [26] approach is used by Zhao and Weng [27] for a cylindrical fiber with an elliptic section in conjunction with Mori–Tanaka’s [25] mean-field theory.

We mention that in the paper [28], the two phases are considered to be isotropic materials. In our work it is considered that the fibers have an anisotropic behavior. This approach represents the contribution of the current investigation to the study of these materials for determining the elastic/viscoelastic constants for this type of composite. Obtaining engineering constants as close as possible to reality is of particular importance in practical applications. Applying the Mori–Tanaka method, it is possible to calculate all the necessary modules for characterizing an orthotropic material, in particular for characterizing the transversely isotropic materials. The compliance matrix can be obtained and allows an experimental verification of the results, by obtaining the creep curves.

Let us now consider a two-phase composite reinforced with fibers uniformly dispersed in the matrix material. We denote a comparison material with (CM). Consider both of the RVE, the real composite and the CM, subjected to the same boundary traction $\bar{\sigma }$. Boundary traction represents a traction applied at the end (boundary surface) of the RVE. The loading condition for both, the RVE and the real composite is the same, a traction noted $\bar{\sigma }$. In the following, we will denote by $C_{m}$ the elastic coefficients matrix and by $C_{f}$ the elastic coefficients of the fiber. It is obvious that under the traction $\bar{\sigma }$, the average strain field in the matrix differs from the average strain in (CM), where the mean stress is $\bar{\sigma }$. Let $\tilde{\varepsilon }$ represent the difference between the average values of the strain. The same thing happens with the stress field. Between the two kinds of material exists a different mean stress $\tilde{\sigma }$. We can conclude:

In CM, the relation between the mean strain field ε° and the mean stress field $\bar{\sigma }$ is:

(1) $$\bar{\sigma }=C_{m}\varepsilon^{0}$$

The mean strain fields in the RVE of the composite are $\varepsilon^{m}=\varepsilon^{0}+\bar{\varepsilon }$ and the mean stress field is $\sigma^{m}=\bar{\sigma }+\tilde{\sigma }$. Consequently, it results

(2) $$\sigma^{m}=\bar{\sigma }+\tilde{\sigma }=C_{m}(\varepsilon^{0}+\bar{\varepsilon })$$

The mean strain field in the fiber differs from that in the matrix through an additional term $\varepsilon^{pt}$ and hence $\varepsilon^{f}=\varepsilon^{m}+\varepsilon^{pt}=\varepsilon^{0}+\tilde{\varepsilon }+\varepsilon^{pt}$. A similar mean stress field differs by the term $\sigma^{pt}$ and therefore, $\sigma^{f}=\bar{\sigma }+\tilde{\sigma }+\sigma^{pt}$. The stress–strain relation becomes:

(3) $$\sigma^{f}=\bar{\sigma }+\tilde{\sigma }+\sigma^{pt}=C_{f}(\varepsilon^{0}+\tilde{\varepsilon }+\varepsilon^{pt})$$

or:

(4) $$\sigma^{f}=\bar{\sigma }+\tilde{\sigma }+\sigma^{pt}=C_{f}(\varepsilon^{0}+\tilde{\varepsilon }+\varepsilon^{pt})=C_{m}(\varepsilon^{0}+\tilde{\varepsilon }+\varepsilon^{pt}-\varepsilon^{*})$$

where the following relation holds:

(5) $$\varepsilon^{pt}=P\varepsilon^{*}$$

In Appendix A, the Eshelby’s transformation tensor *P* for our case is presented, where the symmetry property *P_ikjl_ = P_jikl_ = P_ijtk_* is valid.

To compute the average stress in the whole RVE, the well-known relation as shown below is used:

(6) $$\begin{array}{l} \bar{\sigma }=v_{f}\sigma_{f}+v_{m}\sigma_{m}=v_{f}(\bar{\sigma }+\tilde{\sigma }+\sigma^{pt})+v_{m}(\bar{\sigma }+\tilde{\sigma })\\ =(v_{f}+v_{m})\bar{\sigma }+(v_{f}+v_{m})\tilde{\sigma }+v_{f}\sigma^{pt}=\bar{\sigma }+\tilde{\sigma }+v_{f}\sigma^{pt} \end{array}$$

which, in our case, reduces to:

(7) $$\tilde{\sigma }=-v_{f}\sigma^{pt}$$

For the strain, using a similar procedure, it results in:

(8) $$\bar{\varepsilon }=-v_{f}(\varepsilon^{pt}-\varepsilon^{*})=-v_{f}(P\varepsilon^{*}-\varepsilon^{*})=-v_{f}(P-I)\varepsilon^{*}$$

Hereby *I* is denoted as the unit tensor.

Introducing (7) into (4) yields:

(9) $$C_{f}\left[\varepsilon^{0}-v_{f}\left(P-I\right)\varepsilon^{*}+P\varepsilon^{*}\right]=C_{m}\left[\varepsilon^{0}-v_{f}\left(P-I\right)\varepsilon^{*}+P\varepsilon^{*}-\varepsilon^{*}\right]$$

which after preliminary calculation reduces to:

(10) $$\left[C_{f}\left(-v_{f}\left(P-I\right)+P\right)+C_{m}\left(v_{f}\left(P-I\right)-P+I\right)\right]\varepsilon^{*}+\left(C_{f}-C_{m}\right)\varepsilon^{0}=0$$

or:

(11) $$\left[C_{f}\left(v_{m}P+v_{f}I\right)-C_{m}v_{m}(P-I)\right]\varepsilon^{*}+\left(C_{f}-C_{m}\right)\varepsilon^{0}=0$$

and:

(12) $$\left[v_{m}\left(C_{f}-C_{m}\right)P+v_{f}\left(C_{f}-C_{m}\right)+C_{m}\right]\varepsilon^{*}+\left(C_{f}-C_{m}\right)\varepsilon^{0}=0$$

Finally, it reduces to:

(13) $$\left[\left(C_{f}-C_{m}\right)\left(v_{m}P+v_{f}I\right)+C_{m}\right]\varepsilon^{*}+\left(C_{f}-C_{m}\right)\varepsilon^{0}=0$$

from where:

(14) $$\begin{array}{l} \varepsilon_{11}^{*}=\frac{1}{A}\left(A_{11}\varepsilon_{11}^{o}+A_{12}\varepsilon_{22}^{o}+A_{13}\varepsilon_{33}^{o}\right)\;;\;\\ \varepsilon_{11}^{*}=\frac{1}{A}\left(A_{21}\varepsilon_{11}^{o}+A_{22}\varepsilon_{22}^{o}+A_{23}\varepsilon_{33}^{o}\right)\;;\\ \varepsilon_{11}^{*}=\frac{1}{A}\left(A_{31}\varepsilon_{11}^{o}+A_{32}\varepsilon_{22}^{o}+A_{33}\varepsilon_{33}^{o}\right)\;. \end{array}$$

The coefficients $A_{ij}$ can be found in Appendix A [11]. Using the definitions for the shear strain, we have [11]:

(15) $$\varepsilon_{12}^{*}=\frac{\left(G_{12,f}-G_{m}\right)}{\left(G_{12,f}-G_{m}\right)\left(2v_{m}P_{1212}+v_{f}\right)+G_{m}}\varepsilon_{12}^{0}$$

(16) $$\varepsilon_{23}^{*}=\frac{\left(G_{23,f}-G_{m}\right)}{\left(G_{23,f}-G_{m}\right)\left(2v_{m}P_{2323}+v_{f}\right)+G_{m}}\varepsilon_{23}^{0}$$

(17) $$\varepsilon_{31}^{*}=\frac{\left(G_{31,f}-G_{m}\right)}{\left(G_{31,f}-G_{m}\right)\left(2v_{m}P_{3131}+v_{f}\right)+G_{m}}\varepsilon_{31}^{0}$$

Equations (13)–(17) shown above will be utilized to determine the elastic/viscoelastic coefficients of an RVE considered as an orthotropic body. The results will be applied to a composite reinforced with graphite/epoxy fibers, while this material is considered as transversely isotropic. 

To determine the longitudinal Young’s modulus $E_{m}$ of an orthotropic body, we subject to a pure traction ${\bar{\sigma }}_{11}$ the studied composite and the comparison material. Then, it follows that ${\bar{\sigma }}_{11}=E_{11}{\bar{\varepsilon }}_{11}$ for the composite and ${\bar{\sigma }}_{11}=E_{m}{\bar{\varepsilon }}_{11}^{0}$; ${\bar{\varepsilon }}_{22}^{0}={\bar{\varepsilon }}_{33}^{0}=-\nu_{m}{\bar{\varepsilon }}_{11}^{0}$ if the comparison material is considered.

Using rel. (13) we have:

(18) $$\begin{array}{l} {\bar{\varepsilon }}_{11}={\bar{\varepsilon }}_{11}^{0}+v_{f}{\bar{\varepsilon }}_{11}^{*}={\bar{\varepsilon }}_{11}^{0}+v_{f}\left(\frac{A_{11}}{A}{\bar{\varepsilon }}_{11}^{0}+\frac{A_{12}}{A}{\bar{\varepsilon }}_{22}^{0}+\frac{A_{13}}{A}{\bar{\varepsilon }}_{33}^{0}\right)\\ \;\;\;\;\;\;\;\;\;\;\;\;\;\;\;\;\;\;\;\;\;={\bar{\varepsilon }}_{11}^{0}\left(1+v_{f}a_{11}\right)-v_{f}a_{12}v_{m}{\bar{\varepsilon }}_{11}^{0}-v_{f}a_{13}v_{m}{\bar{\varepsilon }}_{11}^{0}\\ \;\;\;\;\;\;\;\;\;\;\;\;\;\;\;\;\;\;\;\;\;={\bar{\varepsilon }}_{11}^{0}\left[1+v_{f}\left[a_{11}-v_{m}\left(a_{12}+a_{13}\right)\right]\right] \end{array}$$

where: $a_{ij}^{}=A_{ij}^{}/A$, $A_{ij}^{}$ and A are defined in Appendix A; see rel. (A6).

It follows that:

(19) $$E_{11}=\frac{{\bar{\varepsilon }}_{11}^{0}}{{\bar{\varepsilon }}_{11}}E_{m}=\frac{E_{m}}{1+v_{f}\left[a_{11}-v_{m}\left(a_{12}+a_{13}\right)\right]}$$

Similarly, the Young moduli are obtained for the other directions:

(20) $$E_{22}=\frac{{\bar{\varepsilon }}_{22}^{0}}{{\bar{\varepsilon }}_{22}}E_{m}=\frac{E_{m}}{1+v_{f}\left[a_{22}-v_{m}\left(a_{21}+a_{23}\right)\right]}$$

and

(21) $$E_{33}=\frac{{\bar{\varepsilon }}_{33}^{0}}{{\bar{\varepsilon }}_{33}}E_{m}=\frac{E_{m}}{1+v_{f}\left[a_{33}-v_{m}\left(a_{31}+a_{32}\right)\right]}$$

To determine the shear moduli, the following relations are used:

(22) $${\bar{\sigma }}_{12}=2G_{12}{\bar{\varepsilon }}_{12\;};\;\;\;\;\;\;\;\;\;\;\;\;\;\;\;\;\;\;{\bar{\sigma }}_{12}=2G_{m}{\bar{\varepsilon }}_{12}^{0}\;\;\;$$

Recall that:

(23) $${\bar{\varepsilon }}_{12}={\bar{\varepsilon }}_{12}^{0}+v_{f}{\bar{\varepsilon }}_{12}^{*}=\varepsilon_{12}^{*}-v_{f}\frac{G_{12,f}-G_{m}}{\left(G_{12,f}-G_{m}\right)\left(2v_{m}P_{1212}+v_{f}\right)+G_{m}}\varepsilon_{12}^{0}$$

Comparing rel. (22) with rel. (23) it is obtained for $G_{12}$:

(24) $$G_{12}=G_{m}\left(1+\frac{v_{f}}{\frac{G_{m}}{G_{12,f}-G_{m}}+2v_{m}P_{1212}}\right)$$

The other shear moduli are obtained using similar procedures:

(25) $$G_{23}=G_{m}\left(1+\frac{v_{f}}{\frac{G_{m}}{G_{23,f}-G_{m}}+2v_{m}P_{2323}}\right)$$

and

(26) $$G_{31}=G_{m}\left(1+\frac{v_{f}}{\frac{G_{m}}{G_{31,f}-G_{m}}+2v_{m}P_{3131}}\right)$$

To obtain the Poisson’s ratio, the following relations are used:

(27) $${\bar{\varepsilon }}_{22}=-v_{m}{\bar{\varepsilon }}_{11};\;\;\;\;\;\;\;\;\;\;\;\;\;\;\;\;{\bar{\varepsilon }}_{22}^{0}=\;{\bar{\varepsilon }}_{33}^{0}=-v_{m}{\bar{\varepsilon }}_{11}^{0}\;$$

Note that:

(28) $${\bar{\varepsilon }}_{11}={\bar{\varepsilon }}_{11}^{0}+v_{f}{\bar{\varepsilon }}_{11}^{*}={\bar{\varepsilon }}_{11}^{0}+v_{f}a_{11}{\bar{\varepsilon }}_{11}^{0}+v_{f}a_{12}{\bar{\varepsilon }}_{22}^{0}+v_{f}a_{13}{\bar{\varepsilon }}_{33}^{0}={\bar{\varepsilon }}_{11}^{0}\left(1+v_{f}a_{11}\right)+v_{f}a_{12}{\bar{\varepsilon }}_{22}^{0}+v_{f}a_{13}{\bar{\varepsilon }}_{33}^{0}$$

Also

(29) $${\bar{\varepsilon }}_{22}={\bar{\varepsilon }}_{22}^{0}+v_{f}{\bar{\varepsilon }}_{22}^{*}=v_{f}a_{21}{\bar{\varepsilon }}_{11}^{0}+{\bar{\varepsilon }}_{22}^{0}\left(1+v_{f}a_{22}\right)+v_{f}a_{23}{\bar{\varepsilon }}_{33}^{0}$$

or:

(30) $${\bar{\varepsilon }}_{11}=\left[\left(1+v_{f}a_{11}\right)-v_{f}a_{12}v_{m}-v_{f}a_{13}v_{m}\right]{\bar{\varepsilon }}_{11}^{0}=\left[v_{f}a_{21}-v_{m}\left(1+v_{f}a_{22}\right)-v_{m}v_{f}a_{23}\right]{\bar{\varepsilon }}_{11}^{0}$$

Now, substitution of (29) and (30) offers:

(31) $$v_{12}=-\frac{{\bar{\varepsilon }}_{22}}{{\bar{\varepsilon }}_{11}}=-\frac{v_{f}a_{21}-v_{m}\left(1+v_{f}a_{22}\right)-v_{m}v_{f}a_{23}}{1+v_{f}a_{11}-v_{f}a_{12}v_{m}-v_{f}a_{13}v_{m}}$$

which can be rearranged to:

(32) $$v_{12}=\frac{v_{m}-v_{f}\left[a_{22}-v_{m}\left(a_{21}+a_{23}\right)\right]}{1+v_{f}\left[a_{11}-v_{m}\left(a_{12}+a_{13}\right)\right]}$$

Similarly, it can be shown that:

(33) $$v_{23}=\frac{v_{m}-v_{f}\left[a_{22}-v_{m}\left(a_{21}+a_{23}\right)\right]}{1+v_{f}\left[a_{33}-v_{m}\left(a_{7}+a_{8}\right)\right]}$$

and

(34) $$v_{31}=\frac{v_{m}-v_{f}\left[a_{33}-v_{m}\left(a_{31}+a_{32}\right)\right]}{1+v_{f}\left[a_{11}-v_{m}\left(a_{12}+a_{13}\right)\right]}$$

The method presented in this paper offers the elastic/viscoelastic constants of the composite through uniquely determined values. This is in contrast to methods mostly used for determining these values, which are the bounded methods, the current approach provides the lower and the upper bounds of those values. The originality of the method is dependent on the fact that the fibers are not isotropic but have an anisotropic or transverse isotropic behavior, a situation that can often occur in technique and applications. The time response of the material is evaluated using Schapery’s constitutive equation [28]. In this way, nonlinear viscoelastic materials can be studied. So, experimental creep curves can be determined and, in this way, the relations obtained in the paper can be verified.

## 3. Testing Facilities and Results

Within the testing program designed to verify the results obtained in the paper, we aimed to design an experimental system that would allow the simultaneous testing of a large number of samples, in different loading conditions and different temperatures.

Most tests have been realized at 23 °C considering the ambient temperature. The relative humidity was controlled in the room where the measurements were performed.

The test period in which the significant results were obtained was 10 h. As a result, tests were performed for all samples for this interval. The experimental setup for creep testing consists of fifteen “single lever” arrangement traction test stations and seven “double lever” arrangement stations with a 10:1 and 25:1 multiplication ratio, respectively [29]. The system is computer controlled and tests on all existing test stations can be performed simultaneously.

The load level for each station is electronically controlled. The creep stress that occurs in the tested specimen is also measured electronically. The system is based on measurements with foreign gages. The installation makes up for the temperature variations that can appear and distort the results.

The disadvantages of this method consist in the high costs of gages taking into account the great number of tests. A strain gage can be used only once. Other disadvantages consist in the great number of fix-up operations, difficulties in adhering the gages, calibration of gages, etc. An alternative is to use extensometers.

**Figure 2:**
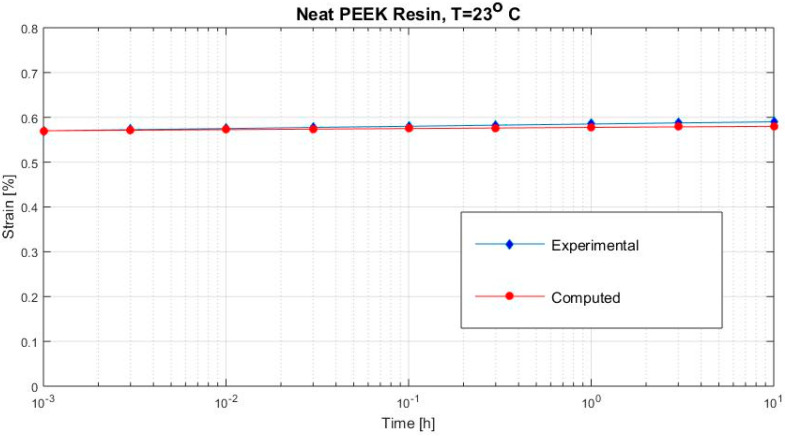
Creep strain (neat polyether-ether-ketone (PEEK) resin subjected to $\sigma_{22}=$ 26 MPa at 23 °C).

**Figure 3:**
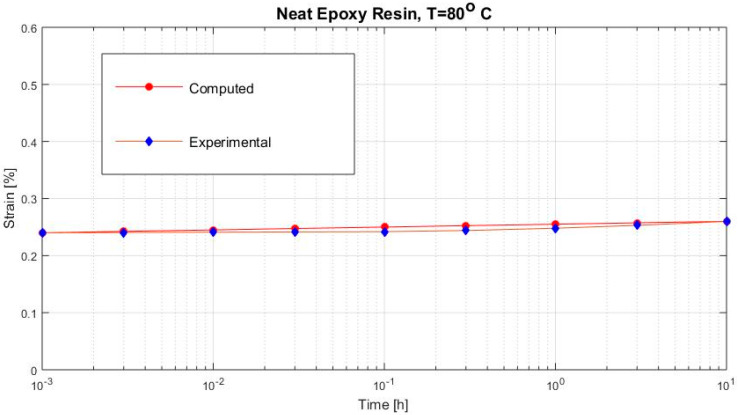
Creep strain (neat epoxy resin loaded with $\sigma_{22}=$ 9 MPa at 80 °C).

**Figure 4:**
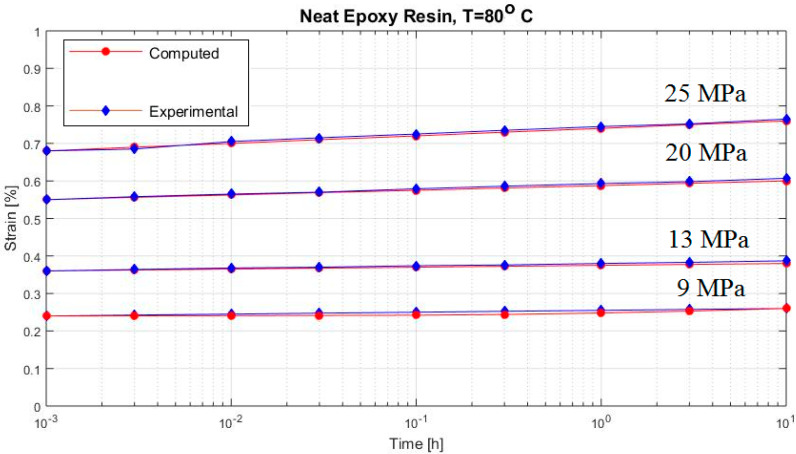
Creep strain (neat epoxy resin at 80 °C).

**Figure 5:**
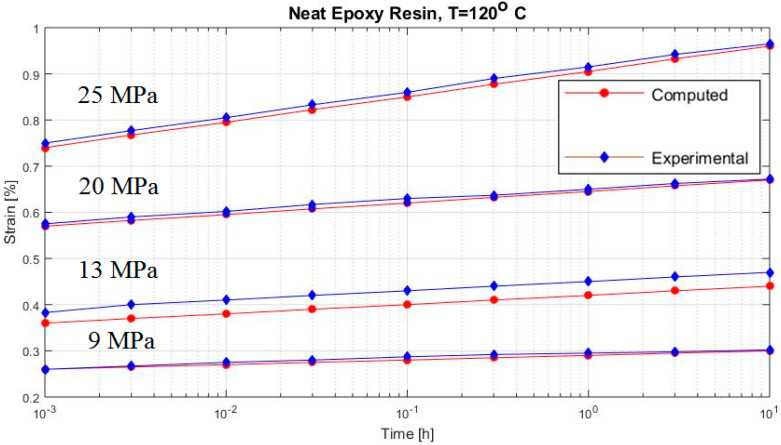
Creep strain (neat epoxy resin at 120 °C).

**Figure 6:**
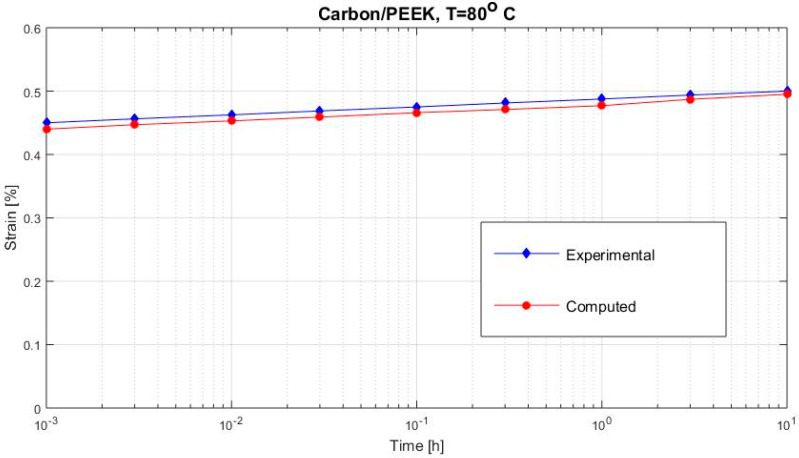
Creep strain $\varepsilon_{22}$ (carbon/PEEK subjected to $\sigma_{22}=$ 36 MPa at 80 °C).

**Figure 7:**
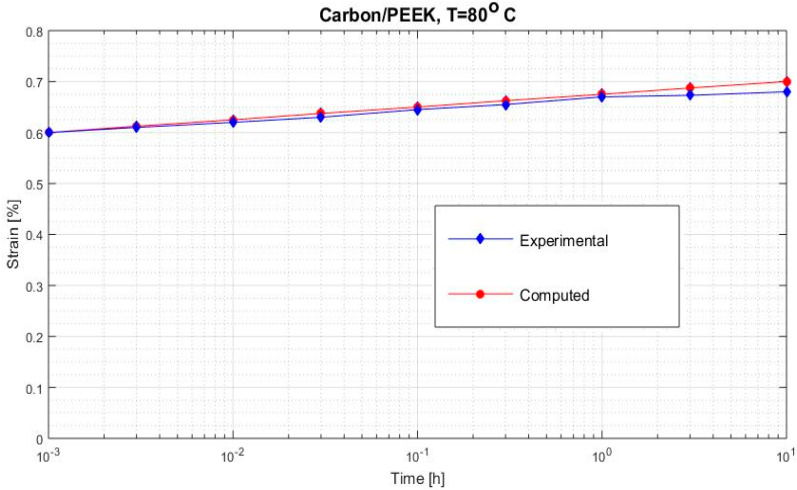
Creep strain $\varepsilon_{22}$ (carbon/PEEK subjected to $\sigma_{22}=$ 48 MPa at 80 °C).

**Figure 8:**
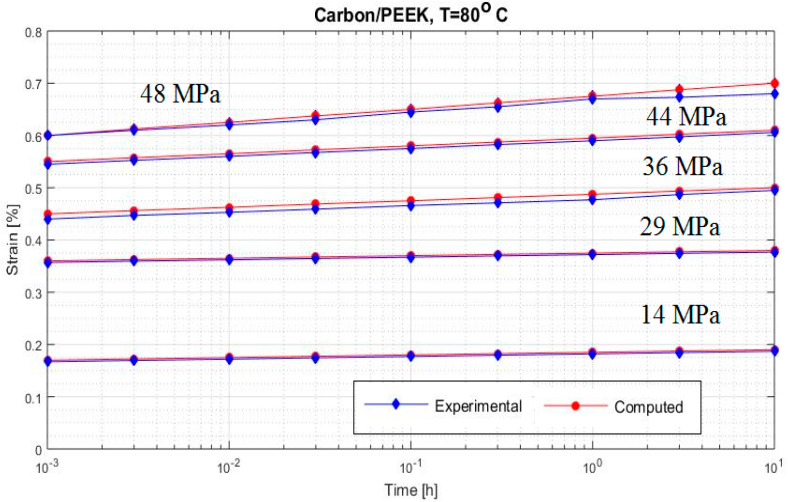
Creep strain $\varepsilon_{22}$ (carbon/PEEK considering different stress at 80 °C).

**Figure 9:**
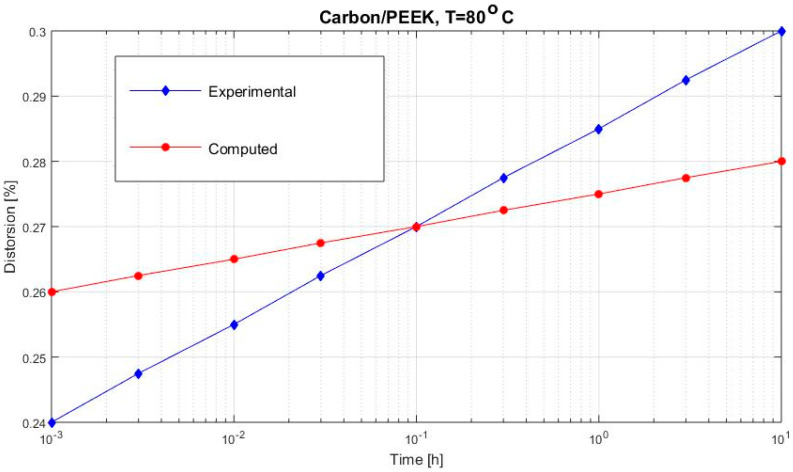
Creep distortion $\gamma_{12}$ (carbon/PEEK subjected to $\tau_{12}=$13 MPa at 80 °C).

**Figure 10:**
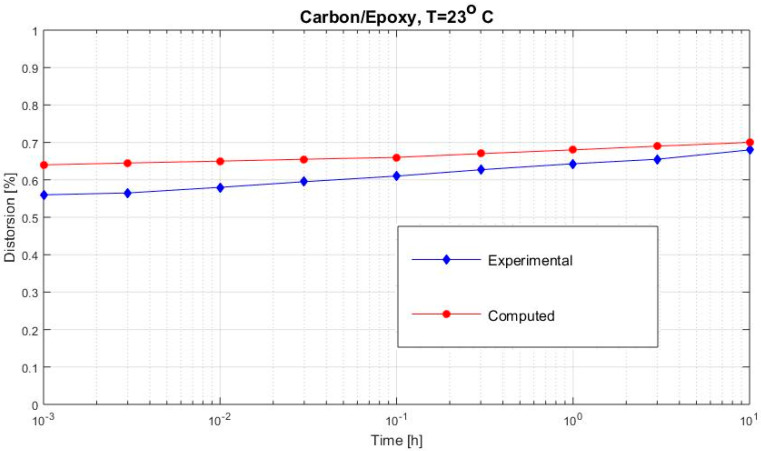
Creep distortion $\gamma_{12}$ (carbon/PEEK subjected to $\tau_{12}=$ 6 MPa at 100 °C).

**Figure 11:**
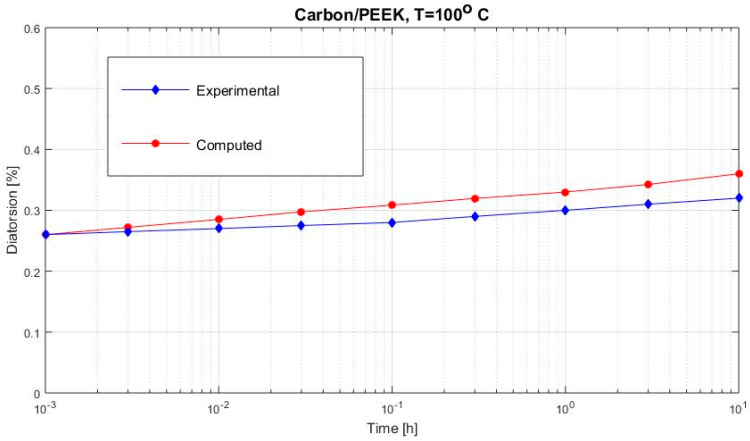
Creep distortion $\gamma_{12}$ (carbon/epoxy subjected to $\tau_{12}=$ 29 MPa at 23 °C).

**Figure 12:**
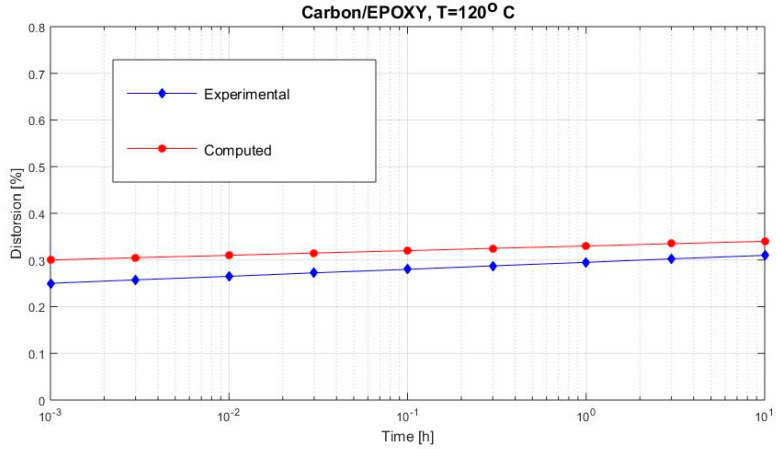
Creep distortion $\gamma_{12}$ (carbon/epoxy subjected to $\tau_{12}=$ 12 MPa at 120 °C).

Figure 2, Figure 3, Figure 4, Figure 5, Figure 6, Figure 7, Figure 8, Figure 9, Figure 10, Figure 11 and Figure 12 show creep curves for the investigated two-phase composite with an isotropic viscoelastic matrix and transversally isotropic fibers. It is easily seen that fair to good prediction of the experimental data is possible with the theoretical procedure introduced in this section. Further discussion of the results in terms of accuracy and comparison with experimental data is presented in the Conclusion section. The nonlinear viscoelastic characterization was established by testing composites at different applied stresses and test temperature. Five stress levels ranging between 10 and 70% were applied. Each of these stresses was tested at four temperatures, while lowering the level of applied stress with increasing temperature. Some of the obtained results are presented in the following figures. The creep behavior of polyether-ether-ketone (PEEK) and epoxy resin laminates are presented. In these cases, the time response obtained with the proposed relations agrees very well with the experimental results. A study of the diagram of the transverse strain $\varepsilon_{22}$ shows a very accurate prediction of mechanical constants obtained at a relatively high temperature of 120 °C. Figure 5 shows that the deviation between the theoretical and experimental results is less than 8%.

The creep curves of the epoxy resin; more values of load at 80 °C are presented in Figure 4 and at 120 °C in Figure 5.

## 4. Conclusions

In the first part of our study we have established theoretical values of some mechanical characteristics of a composite material with two components. In this paper it was considered that the reinforcing fibers are homogeneous and transversely isotropic. The matrix is isotropic. With these hypotheses, a prediction was made of the values of elastic/viscoelastic constants of a biphasic composite material depending on the values of elastic constants of the constituent materials [30]. The method applied in the paper used the field method of Mori–Tanaka, extended into the visco-elastic domain to determine the time response of the composite and creep curves [31,32,33,34,35,36,37,38,39,40]. Thus, experimental results can be obtained to validate the theoretical results obtained. A good agreement was obtained between experimental and predicted results for pure resin and 90 degree test specimens.

The same result was not obtained for the 45 degree specimen, where the differences are slightly larger, although they are also very close to the theoretical results. In order to increase the precision, it is proposed to use more precise models, which should take into account the change of the orientation of the fibers, especially at high values of applied tension and temperature.

Creep tests were performed in the current study to determine the nonlinear behavior of neat and carbon fiber-reinforced polyether-ether-ketone (PEEK) and epoxy resin, on {90}]_4s_ and {±45}_4s_ laminates. The same laminates were used to study the transverse properties and shear properties.

The number of experiments in which the temperature, the applied stress and the combination of materials varied was large enough, after comparing the experimental results with the theoretical ones, to provide confidence in the calculation formulas obtained [4].

## Appendix A

Eshelby’s Tensor for an Elliptic Cylinder

The Eshelby’s $P_{ijkl}$ tensor written for fibers with an elliptical cross section has the components [28]

(A1) $$P_{2222}=\frac{1}{2\left(1-v_{m}\right)}\left[\frac{1+2\alpha }{{\left(1+\alpha \right)}^{2}}+\frac{1-2v_{m}}{1+\alpha }\right]\;P_{3333}=\frac{\alpha }{2\left(1-v_{m}\right)}\left[\frac{\alpha +2}{{\left(1+\alpha \right)}^{2}}+\frac{1-2v_{m}}{1+\alpha }\right]\;P_{2211}=\frac{v_{m}}{1-v_{m}}\frac{1}{1+\alpha }\;P_{2233}=\frac{1}{2\left(1-v_{m}\right)}\left[\frac{1}{{\left(1+\alpha \right)}^{2}}+\frac{1-2v_{m}}{1+\alpha }\right]\;P_{3311}=\frac{v_{m}}{1-v_{m}}\frac{\alpha }{1+\alpha }\;P_{3322}=\frac{\alpha }{2\left(1-v_{m}\right)}\left[\frac{\alpha }{{\left(1+\alpha \right)}^{2}}+\frac{1-2v_{m}}{1+\alpha }\right]\;P_{1212}=\frac{1}{2\left(1+\alpha \right)}\;P_{1313}=\frac{\alpha }{2\left(1+\alpha \right)}\;P_{2323}=\frac{1}{4\left(1-v_{m}\right)}\left[\frac{1+\alpha^{2}}{{\left(1+\alpha \right)}^{2}}+\left(1-2v_{m}\right)\right]$$

and

(A2) $$P_{1111}=0\;\;;\;\;P_{1122}=0\;\;\;;\;\;\;\;P_{1133}=0$$

where all other $P_{ijkl}=0$. Relation (9) offers the liaison between $\varepsilon_{ij}^{0}$ and $\varepsilon_{ij}^{*}$. For this it is necessary to know that: (see Reference [25]).

(A3) $$\left[\begin{array}{ccc} M_{1} & M_{2} & M_{3}\\ M_{4} & M_{5} & M_{6}\\ M_{7} & M_{8} & M_{9} \end{array}\right]\left\{\begin{array}{c} \varepsilon_{11}^{*}\\ \varepsilon_{22}^{*}\\ \varepsilon_{33}^{*} \end{array}\right\}+\left[\begin{array}{ccc} N_{1} & 1 & 1\\ 1 & N_{1} & 1\\ 1 & 1 & N_{1} \end{array}\right]\left\{\begin{array}{c} \varepsilon_{11}^{0}\\ \varepsilon_{22}^{0}\\ \varepsilon_{33}^{0} \end{array}\right\}=0$$

where

(A4) $$M_{1}=v_{f}N_{1}+N_{2}+v_{m}\left(P_{2211}+P_{3311}\right)\;M_{2}=v_{f}+N_{3}+v_{m}\left(P_{2222}+P_{3322}\right);\;M_{3}=v_{f}+N_{3}+v_{m}\left(P_{2233}+P_{3333}\right);\;M_{4}=v_{f}+N_{3}+v_{m}\left(P_{2211}+P_{3311}\right)\;;\;M_{5}=v_{f}N_{1}+N_{2}+v_{m}\left(N_{1}P_{2222}+P_{3322}\right)\;M_{6}=v_{f}+N_{3}+v_{m}\left(P_{2233}+P_{3333}\right);\;M_{7}=v_{f}+N_{3}+v_{m}\left(N_{1}P_{3311}+P_{2211}\right);\;M_{8}=v_{f}+N_{3}+v_{m}\left(N_{1}P_{3322}+P_{2222}\right);\;M_{9}=v_{f}N_{1}+N_{2}+v_{m}\left(P_{3333}+P_{2233}\right)$$

with the following expressions for $N_{i}$:

(A5) $$N_{1}=1+2\left(G_{f}-G_{m}\right)/\left(\lambda_{f}-\lambda_{m}\right);\;N_{2}=\left(\lambda_{m}+2G_{m}\right)/\left(\lambda_{f}-\lambda_{m}\right)\;N_{3}=\lambda_{m}/\left(\lambda_{f}-\lambda_{m}\right)$$

where $\lambda_{f}$ and $\lambda_{m}$ are the Lame’s constants, respectively, for the fiber matrix.

From (A3) it results:

(A6) $$\left\{\begin{array}{c} \varepsilon_{11}^{*}\\ \varepsilon_{22}^{*}\\ \varepsilon_{33}^{*} \end{array}\right\}=\frac{1}{A}\left[\begin{array}{ccc} A_{11} & A_{12} & A_{13}\\ A_{21} & A_{22} & A_{23}\\ A_{31} & A_{32} & A_{33} \end{array}\right]\left\{\begin{array}{c} \varepsilon_{11}^{0}\\ \varepsilon_{22}^{0}\\ \varepsilon_{33}^{0} \end{array}\right\}$$

(A7) $$A_{11}=A\cdot a_{11}=N_{1}\left(M_{6}M_{8}-M_{5}M_{9}\right)+M_{3}\left(M_{5}-M_{8}\right)+M_{2}\left(M_{9}-M_{6}\right);\;A_{12}=A\cdot a_{12}=N_{1}\left(M_{2}M_{9}-M_{3}M_{8}\right)+M_{6}\left(M_{8}-M_{2}\right)+M_{5}\left(M_{3}-M_{9}\right);\;A_{13}=A\cdot a_{13}=N_{1}\left(M_{3}M_{5}-M_{2}M_{6}\right)+M_{8}\left(M_{6}-M_{3}\right)+M_{9}\left(M_{2}-M_{5}\right);\;A_{21}=A\cdot a_{21}=N_{1}\left(M_{4}M_{9}-M_{6}M_{7}\right)+M_{1}\left(M_{6}-M_{9}\right)+M_{3}\left(M_{7}-M_{4}\right);\;A_{22}=A\cdot a_{22}=N_{1}\left(M_{3}M_{7}-M_{1}M_{9}\right)+M_{4}\left(M_{9}-M_{3}\right)+M_{6}\left(M_{1}-M_{7}\right);\;A_{23}=A\cdot a_{23}=N_{1}\left(M_{1}M_{6}-M_{3}M_{4}\right)+M_{9}\left(M_{4}-M_{1}\right)+M_{7}\left(M_{3}-M_{6}\right);\;A_{31}=A\cdot a_{31}=N_{1}\left(M_{5}M_{7}-M_{4}M_{8}\right)+M_{2}\left(M_{4}-M_{7}\right)+M_{1}\left(M_{8}-M_{5}\right);\;A_{32}=A\cdot a_{32}=N_{1}\left(M_{1}M_{8}-M_{2}M_{7}\right)+M_{5}\left(M_{7}-M_{1}\right)+M_{4}\left(M_{2}-M_{8}\right);\;A_{33}=A\cdot a_{33}=N_{1}\left(M_{2}M_{4}-M_{1}M_{5}\right)+M_{7}\left(M_{5}-M_{2}\right)+M_{8}\left(M_{1}-M_{4}\right)$$

## References

[1] Hashin Z., Shtrikman S. (1962). On Some Variational Principles in Anisotropic and Non-homogeneous Elasticity. J. Mech. Phys. Solids.

[2] Hashin Z., Shtrikman S. (1963). A Variational Approach to the Theory of the Elastic Behavior of Multiphase Materials. J. Mech. Phys. Solids.

[3] Brüller O., Katouzian M., Vlase S. (1994). Some Results on the Nonlinear Viscoelastic Behaviour of Fiber Reinforced Compozite. Congres—Recent Advances in Experimental Mechanics.

[4] Katouzian M., Bruller O.S., Horoschenkoff A. (1995). On the Effect of Temperature on the Creep Behavior of Neat and Carbon Fiber Reinforced PEEK and Epoxy Resin. J. Compos. Mater..

[5] Budiansky B. (1965). On the elastic moduli of some heterogeneous materials. J. Mech. Phys. Solids.

[6] Chen T., Dvorak G.J., Benveniste Y. (1992). Mori-Tanaka Estimates of the Overall Elastic Moduli of Certain Composite Materials. J. Appl. Mech..

[7] Cherkaev A., Gibiansky L. (1993). Coupled estimates for the bulk and shear moduli of a two-dimensional isotropic elastic composite. J. Mech. Phys. Solids.

[8] Christensen R. (1969). Viscoelastic properties of heterogeneous media. J. Mech. Phys. Solids.

[9] Hill R. (1964). Theory of Mechanical Properties of Fiber-strengthened Materials: I Elastic Behavior. J. Mech. Phys. Solids.

[10] Hill R. (1964). Theory of Mechanical Properties of Fiber-strengthened Materials: II Inelastic Behavior. J. Mech. Phys. Solids.

[11] Hill R. (1965). Theory of Mechanical Properties of Fiber-strengthened Materials: III Self-Consistent Model. J. Mech. Phys. Solids.

[12] Hashin Z. (1965). On Elastic Behavior of Fibre Reinforced Materials of Arbitrary Transverse Phase Geometry. J. Mech. Phys. Solids.

[13] Niculita C., Vlase S., Bencze A., Mihalcica M., Calin R., Serbina L. (2011). Optimum stacking in a multi-ply laminate used for the skin of adaptive wings. Optoelectron. Adv. Mater. Rapid Commun..

[14] Stanciu A., Teodorescu-Draghicescu H., Vlase S., Scutaru M., Calin R. (2012). Mechanical behavior of CSM450 and RT800 laminates subjected to four-point bend tests. Optoelectron. Adv. Mater. Rapid Commun.

[15] Hashin Z., Rosen B.W. (1964). The Elastic Moduli of Fiber-Reinforced Materials. J. Appl. Mech..

[16] Golub V.P., Pavlyuk Y.V., Reznik V.S. (2020). Analysis of Creep Strains and Stress Relaxation in Thin-Walled Tubular Members Made of Linear Viscoelastic Materials. Superposition of Shear and Volume Creep. Int. Appl. Mech..

[17] Azizi M.A., Ariffin A.K. (2019). Peridynamic model for nonlinear viscoelastic creep and creep rupture of Polypropylene. J. Mech. Eng. Sci..

[18] Wang Z., Smith D.E. (2019). Numerical analysis on viscoelastic creep responses of aligned short fiber reinforced composites. Compos. Struct..

[19] Cheng Y., Li H., Li L., Zhang Y., Wang H., Bai Y. (2019). Viscoelastic Properties of Asphalt Mixtures with Different Modifiers at Different Temperatures Based on Static Creep Tests. Appl. Sci..

[20] Pichler C., Maier M., Lackner R. (2018). Viscoelastic Response of Closed-Cell Polyurethane Foams from Half Hour-Long Creep Tests: Identification of Lomnitz Behavior. J. Eng. Mater. Technol..

[21] Zhang X., Zheng Y., Li G.-Y., Liu Y.-L., Cao Y. (2019). Indentation creep tests to assess the viscoelastic properties of soft materials: Theory, method and experiment. Int. J. Non-Linear Mech..

[22] Vlase S., Marin M., Öchsner A., Scutaru M.L. (2018). Motion equation for a flexible one-dimensional element used in the dynamical analysis of a multibody system. Contin. Mech. Thermodyn..

[23] Khodadadian A., Parvizi M., Abbaszadeh M., Dehghan M., Heitzinger C. (2019). A multilevel Monte Carlo finite element method for the stochastic Cahn–Hilliard–Cook equation. Comput. Mech..

[24] Khodadadian A., Noii N., Parvizi M., Abbaszadeh M., Wick T., Heitzinger C. (2020). A Bayesian estimation method for variational phase-field fracture problems. Comput. Mech..

[25] Mori T., Tanaka K. (1973). Average Stress in the Matrix and Average Elastic Energy of Materials with Misfitting Inclusions. Acta Metall..

[26] Eshelby J.D. (1957). The determination of the elastic field of an ellipsoidal inclusion, and related problems. Proc. R. Soc. Lond. Ser. A Math. Phys. Sci..

[27] Zhao Y.H., Weng G.J. (1990). Effective Elastic Moduli of Ribbon-Reinforced Composites. J. Appl. Mech..

[28] Lou Y.C., Schapery R.A. (1971). Viscoelastic Characterization of a Nonlinear Fiber-Reinforced Plastic. J. Compos. Mater..

[29] Katouzian M., Vlase S., Calin R. (2011). Experimental procedures to determine the viscoelastic parameters of laminated composites. J. Optoelectron. Adv. Mater..

[30] Itu C., Ochsner A., Vlase S., Marin M.I. (2019). Improved rigidity of composite circular plates through radial ribs. Proc. Inst. Mech. Eng. Part L J. Mater. Des. Appl..

[31] Sadeghpour E., Guo Y., Chua D., Shim V.P. (2020). A modified Mori–Tanaka approach incorporating filler-matrix interface failure to model graphene/polymer nanocomposites. Int. J. Mech. Sci..

[32] Song Z., Peng X., Tang S., Fu T. (2020). A homogenization scheme for elastoplastic composites using concept of Mori-Tanaka method and average deformation power rate density. Int. J. Plast..

[33] Barral M., Chatzigeorgiou G., Meraghni F., Léon R. (2020). Homogenization using modified Mori-Tanaka and TFA framework for elastoplastic-viscoelastic-viscoplastic composites: Theory and numerical validation. Int. J. Plast..

[34] Rajabi J., Mohammadimehr M. (2019). Bending analysis of a micro sandwich skew plate using extended Kantorovich method based on Eshelby-Mori-Tanaka approach. Comput. Concr. Int. J..

[35] Pan J., Bian L. (2019). A re-formulation of the Mori–Tanaka method for predicting material properties of fiber-reinforced polymers/composites. Colloid Polym. Sci..

[36] Ogierman W. (2019). Hybrid Mori-Tanaka/Finite Element Method in Homogenization of Composite Materials with Various Reinforcement Shape and Orientation. Int. J. Multiscale Comput. Eng..

[37] Tran V., Brisard S., Guilleminot J., Sab K. (2018). Mori–Tanaka estimates of the effective elastic properties of stress-gradient composites. Int. J. Solids Struct..

[38] Sadowski P., Kowalczyk-Gajewska K., Stupkiewicz S. (2017). Consistent treatment and automation of the incremental Mori–Tanaka scheme for elasto-plastic composites. Comput. Mech..

[39] Aragh B.S., Batra R., Mansur W., Peters F. (2017). Thermal response of ceramic matrix nanocomposite cylindrical shells using Eshelby-Mori-Tanaka homogenization scheme. Compos. Part. B Eng..

[40] Marin M., Vlase S., Paun M. (2015). Considerations on double porosity structure for micropolar bodies. AIP Adv..

